# American Medical Schools

**Published:** 1838

**Authors:** 


### REVIEWS

AND

### BIBLIOGRAPHICAL NOTICES.

### Art. IX.—American Medical Schools.

*Report on the Organization and Administration of the Medical Schools of the United States.**

Notwithstanding the *extra limites* of our last number, presented the entire Journal of the late Medical Convention at the Capital of Ohio, we propose to reprint, in this place, one of the reports adopted by that body, for the purpose of discussing some of its propositions. They are as follow:—

1. "*Resolved*, That in the opinion of this Convention, the sessions of the different Medical Schools, throughout the Union, are too short, and that they ought to be extended one month, and the students required to stay to the *end* of the term.

2. *Resolved*, That the number of Professorships is too few, and that ampler provision should be made for teaching Physiology, Pathological Anatomy, Pharmacy, and the Natural History of Medicines, Botany, Comparative Anatomy, Meteorology, Medical Jurisprudence, and Mental Physiology.

3. *Resolved*, That, if practicable, our Medical Schools should be so organized, as that Students, in their first course, would have their attention chiefly directed upon Special Anatomy, Physiology, Chemistry, Pharmacy, and the other elementary branches; and their second upon Pathological Anatomy, Therapeutics, the practice of Physic, Surgery and Obstetrics.

*Proceedings of the Medical Convention of Ohio, at Columbus, January 1838.

4. *Resolved*, That in admitting Candidates to examination for degrees, a stricter examination than is at present shown, should be had to their preliminary education.

5. *Resolved*, That the practice of graduating young men, before they are 21 years of age, should be abandoned.

6. *Resolved*, That no Pupil ought to be graduated before the end of four years, from the time he commenced the study of Medicine.

7. *Resolved*, That, if the various Schools of the Union, were to send representatives to a meeting at some central point, to confer together, many of their existing defects, by a simultaneous, co-operative effort, might be successfully remedied, and that we, respectfully, recommend such a Convention to be held. Till when it would not be practicable, nor should it be expected, that any single institution will attempt the reforms which are here proposed.

8. *Resolved*, That the Corresponding Secretary be instructed to send a printed copy of the Proceedings of this Convention, to all the Medical Institutions of the United States, with a letter, calling the attention of their Professors to these Resolutions."

Before proceeding to comment on these resolutions, we must express our joy, that a portion of the profession, in a primary assembly, have ventured to speak out, and fearlessly indicate some of the existing defects, in the organization, conduct and adminis-tion of our schools.

We regret that the committee charged with this subject, did not prefix to their resolutions, a report on the necessity of what they have recommended. It would have rendered the labors of the Convention, on this point, more effective. Their first resolution, however, contains suggestions in which every reflecting member of the profession must concur. That the lecture terms, in all the schools of the Union, are too short, is undeniable. Why, we would ask, should they be shorter than those of European schools? Is there one science for the old, and another for the new world? Is the acquisitive power of the young man of America, greater than that of the young man of Europe? Is his diligence superior? All these questions, we apprehend, must be answered in the negative—especially the first and last; and, if so, a brief and superficial course of study, can no more make a *good* physician in the United States, than in Scotland, France, or Germany. Wherefore, then, do we persevere in sessions of 16, 13 and even 10 weeks; requiring, an attendance, moreover, on but two of these? It can only be, for the purpose of supplying our people with "cheap goods," for which, our elder brethren over the water, affirm, we have a sort of national *penchant.* With this taste we are not inclined to quarrel, while it is confined to wares and merchandize; but when it extends to scientific qualifications, in the most responsible and difficult of all the professions, we have little disposition to see it gratified. It is criminal to tamper with human life, and those who conduct our medical schools, should not feel themselves at liberty to be accessories to such a crime. When, however, they depress the standard of medical knowledge, to the attainments which can be made in two sessions of a few weeks, they are, in fact, the real authors of the mischief. It is not to be expected, that students, (or, at least, the mass of them,) will find fault with this easy method of becoming *doctors;* and the corrective must be applied by their teachers. Were our sessions extended a month, by a concert of action throughout the Union, our students would quietly acquiesce in the change, and all the ambitious, inquisitive or conscientious among them, would rejoice at the innovation. We would even go further, and carry the extension to two months, the effect of which would be, to diminish the *aggregate* number of practioners, while it greatly increased the proportion of the thoroughly indoctrinated.

But the resolution declares, by implication, that, short as are our sessions, the pupils are not required, and do not see them out. Impatience is an American characteristic; and this trait, instead of being corrected, is directly cherished, by the laxness of our regulations. What can be more absurd, than to see our students dispersing, when a short course of lectures is yet unfinished? And still nothing is more common; for it prevails in all the schools of the Union. We have seen students back in the valley of the Mississippi, while the lectures which they had travelled 700 miles and paid their money to attend, were still going on in an eastern city. This was not the fault of the lectures, but of the lecturers, who did not decree, that credit should not be given for a course, which was not attended to its close. Were this the law of all our institutions, the impatience of our pupils would be abolished, and their acquirements greatly augmented. Every student ought, therefore, to be required to take with him a ticket of valediction, not less than of matriculation; and this, except for *special* reasons, should never be issued till the last valedictory was pronounced.

We cannot but cherish a hope, that this regulation, together with an extension of the term, will, at no distant day, be adopted by all our schools. The effect on the American profession would be instantaneous, and, in all respects, salutary. An immediate elevation of professional character would, indeed, be the consequence, and society as speedily come in for a share of the benefits.

The second resolution furnishes a strong argument in support of the first, and ought, indeed, to have preceded it in the series. It looks directly to the limited range of studies prescribed and pursued, in nearly every school of the Union. Indeed, we may affirm, that there is not one, in which the cycle is as comprehensive, as the nature of the medical profession demands. In most of our institutions, descriptive anatomy, chemistry, materia medica, surgery, obstetrics, and the practice of physic, taken in their ancient and restricted limits, are nearly all that is taught. But, if a student be not well instructed in general anatomy, physiology and morbid anatomy, how can he understand pathology, and the principles of surgery? If he do not know the physiology of the mind, how can he successfully study its diseased manifestations? If he be ignorant of botany, organic chemistry, and pharmacutic manipulations, to say nothing of mineralogy and geology, does he not come up to the study of materia medica and the art of prescribing, in a state of most hopeless deficiency? How can a teacher of the theory and practice, discuss the orgin of many large groups of diseases, before students, who are unacquainted with the laws and operations of the atmosphere? Finally, with what success can a physician acquit himself, as a witness, before a judicial tribunal, if he be ignorant of medical jurisprudence?

Each of these questions suggests its own answer. In truth, the branches here enumerated, should be regarded as essential links, in the chain of professional studies; and without a better knowledge of them, than can be had in *any* of our schools, the student cannot be regarded as adequately prepared for the duties of his profession. Several of these sciences, or new applications of science, are of modern development, and show us, that with the progress of discovery and invention, the labors of the medical student are increased, not diminished. Some of them require separate professorships, or, at least, lectureships; and hence the number of chairs in our institutions ought to be augmented, and with this augmentation should come lengthened sessions; that our students may not be oppressed by too many lectures daily.

Of the third resolution we scarcely know how to speak. In theory, it is undoubtedly correct; but how, in this case, can practice be made to accord with principle? It is not only absurd, but actually injurious, for the student who has recently commenced the study of medicine, and is not yet acquainted with the structure and functions of the body, with chemistry, or the rudiments of botany and zoology, to engage in the high and difficult inquiries of pathology and practical medicine; and, still, in the present organization of our schools, this is constantly done. The *beau ideal* of collegiate medical instruction, would be for students, in their first course, to devote themselves to anatomy—special, general and pathological, with dissections; to physiology—corporeal and mental; to chemistry, pharmacy, and the classification of medicines; and to so much of the history of the mineral, vegetable and animal kingdoms, as is necessary to a due understanding of the two last;—and in the second session, to give their chief attention to therapeutics, symptomatology, ætiology, practice, surgery and obstetrics. To do this, it would be necessary to class the pupils; an object, which, however desirable, would be of difficult accomplishment, for several reasons. First, a large number of them design (most improperly) to go into practice at the end of one course of lectures, and, therefore, wish to attend the whole. Second, each of them comes to the school, very differently advanced in the different branches, so that scarcely one would be willing to confine himself to the lower, and quite as few would be prepared to enter the higher class. Third, they visit medical schools to hear lectures—not to hear and *read* also; and would not, therefore, be satisfied with three or four a day; or, if they should, the true reason with many of them would be, that it left them more time for listlessness and recreation. It is greatly to be regretted, that private preceptors do not confine the reading of their pupils, in the early period of their studies, to the introductory branches, and send them, as soon as they have taken a bird's-eye view, and become somewhat familiar with technical terms, to a medical school; with instruction to limit themselves to the lectures which are proper for the first session. After attending *it*, they should engage in a course of more practical studies, and then return to the university for graduation. On this subject, the report of Dr. Gans, in the Proceedings of the Convention, contains some very sensible recommendations. It is to be feared, however, that for a long time to come, our brethren, who do not reside in the immediate neighborhood of medical schools, will think, or at least *act*, differently from what is here advised; and equally to be apprehended, that those who prescribe the policy of our institutions, will neglect the establishment of junior and senior classes. Meanwhile, we may hope, however, that the students themselves, will become more and more deeply impressed with the importance of devoting the first session chiefly to the elementary branches, and the second, to the practical.

The fourth resolution, relating to the defective *preliminary* education of students of medicine, presents a subject of the deepest possible interest, especially in the Western States. The first cause of the whole difficulty was the abolition of the requirement, that inaugural theses should be written in Latin. But this abolition itself had a cause, which was the limited cultivation of classical learning in the United States, and the general deficiency of good seminaries. If Latin theses had still been required, none who had not made respectable elementary attainments, would have been put to the study of medicine. Such a restriction, it must be confessed, would have kept many from the study of medicine who might have become useful, and even eminent, but it would have excluded a much larger number, whose preparatory education and early mental discipline, were totally inadequate; and who never overcame these disadvantages. Their places, moreover, would have been occupied by candidates for the profession, better prepared in these respects,who have been turned into other pursuits, by the crowd which has been invited to the temple of medicine. When any and every mouth could utter the magical "open sessame," enough would always be found to pronounce it. The resolution before us seems to intimate, that the remedy for this evil is with our schools; but, we rather think it lies as much with private as public preceptors; indeed, still more, for if the former would not receive unprepared young men as students, they would not be thrown on the latter. The recommendation is, however, a good one; and if acted on with vigilance and firmness, both young men and the physicians with whom they propose to study, would soon learn the necessity of a more careful literary preparation.

The fifth resolution refers to the practice, prevalent, more or less, in all our schools, of graduating candidates before they are 21 years of age. Now, we have no doubt, that many youth make greater attainments by 19, than others by 25; but attainments are not all that is requisite to a correct and successful practice of the profession. A ripe and sound judgment, is equally indispensable, and this is seldom acquired, even by 21. If the rule of not granting diplomas, unless the candidate had arrived at that age, were rigidly observed, which we know is not the case, our youth would prolong their *scholastic* studies, (a great *desideratum*,) and not come up to the practical duties of the profession, till their minds had acquired such maturity, as would enable them to use their knowledge with judgment and effect.

The sixth resolution is connected with the last. It expresses as the opinion of the Convention, that four years of pupilage should be required, as a condition to graduation. What will those physicians think of this, who encourage or permit their students to go into practice at the end of 18 or 24 months, without having, perhaps, even attended one course of lectures during that brief period! A frightful number do this—and apparently feel as little compunction, as the self-conceited stripling, who knows not his own defects, and is even unable, afterwards, to appreciate their sad consequences, to the honor of the profession or the happiness of society. For ourselves, we do not hesitate to say, that the universal and unalterable law should be—*at least* FOUR *years of study*. We may be told, however, that but few of our students have the pecuniary means of continuing their studies throughout such a period. But can this change the principle of duty, or the nature of things? They who have not the means of qualifying themselves for a vocation, should not attempt it. The advancement of the science, and the *able* and faithful discharge of its practical duties, should be regarded as objects of higher importance, than the gratification of a desire, in any individual, to belong to a profession, for which, unfortunately, he may not have the means necessary to prepare himself. We know full well how to sympathize with the destitute in means, having ourselves been of that *genus;* but we know, also, something of the extent and depth of scientific acquirement, which the practical duties of physic and surgery demand, and cannot, conscientiously, grant, that more is due to the feelings of a young man, than to the dignity of the profession, and the welfare of the community united.

The last resolution suggests, that if the various schools of the Union, would send delegates to a Convention, in some central city, they might devise correctives for many of the imperfections, in their organization and administration, which now exist. This could, undoubtedly, be done. Philadelphia is the proper place for such a meeting, and the University of Pennsylvania, should appoint the time and give the invitation. We cannot doubt, that it would be attended by delegates from every institution in the Union. In such a Convention, a multitude of subjects of the deepest interest, might be discussed, and many reforms and improvements, not merely suggested, but their simultaneous execution secured. As the charters of our schools are enacted by different sovereign states, they have no other connection with each other, than that which results from the national compact of those states. Hence the franchises granted are different, and much diversity in organization, requirement and fees of tuition, exists among them—excluding that uniformity, which would throw competition for pupils entirely on the talents of the professors, and the means provided for illustrating their lectures. No one denies, that the best of our institutions are defective, and that some of them must, from numerous imperfections (not personal) *ex necessitate*, send forth many superficial and incompetent *alumni*. It is high time that a remedy was applied; but how can this be done, without community and concert of action? In a country where a limited course of study, short sessions, and low fees, are so consonant to the taste of the majority of students, no change, for the better, is likely to be made in any of them, except it be simultaneous and general. Each institution will apprehend evil consequences to itself, should it advance beyond the rest; but a joint and common movement, would keep all in the same comparative state, while it elevated the whole—and with them, the science, dignity and usefulness of the profession.

We earnestly and respectfully commend this subject, to all who are in stations that will permit them to act upon it. The results anticipated by the Convention from the measure proposed by them, do not appear to us either visionary or exaggerated. We would, also, appeal to our brethren of the periodical press, and exhort them to put forth their power. It is but a part of the duty and end of that press, to publish and republish matters that are purely scientific. The provisions made for the education of those who are to practise, and improve or deteriorate the science, should be taken up and fearlessly but courteously discussed. The American profession is yet in its forming stage, and requires for its full and beneficial organization, that the medical press should disseminate far and wide, its suggestions, opinions and strictures. The eight journals of the Union, are capable of wielding a powerful influence—they should feel their strength, and direct it against the causes, which impede the advancement of the profession. D.

